# The Effect of Dextran Sulfate—as Model Glycosaminoglycan Analogue—on Membrane Lipids: DPPC, Cholesterol, and DPPC–Cholesterol Mixture. The Monolayer Study

**DOI:** 10.1007/s00232-018-0041-z

**Published:** 2018-07-20

**Authors:** Katarzyna Makyła-Juzak, Anna Chachaj-Brekiesz, Patrycja Dynarowicz-Latka, Paweł Dąbczyński, Joanna Zemla

**Affiliations:** 1 Department of General Chemistry, Faculty of Chemistry, Jagiellonian University, Gronostajowa 2, 30-387 Kraków, Poland; 2 Institute of Physics, Jagiellonian University, Łojasiewicza 11, 30-348 Kraków, Poland; 3 Institute of Nuclear Physics, Polish Academy of Sciences, Radzikowskiego 152, 31-342 Kraków, Poland

**Keywords:** Glycosaminoglycans (GAGs), Polysaccharides, Dextran sulfate (DS), Model membrane, Langmuir monolayers

## Abstract

Glycosaminoglycans (GAGs) are essential components of the extracellular matrices (ECMs) located on the outer surface of cellular membranes. They belong to the group of polysaccharides involved in diverse biological processes acting on the surface and across natural lipid membranes. Recently, particular attention has been focused on possible role of GAGs in the amyloid deposits. The amyloid formation is related to a disorder in protein folding, causing that soluble—in normal conditions—peptides become deposited extracellularly as insoluble fibrils, impairing tissue structure and its function. One of the hypothesis holds that GAGs may inhibit amyloid formation by interacting with the lipid membrane by blocking the accumulation of protein aggregates on the membrane surface. Although the biophysical properties of GAGs are described rather well, little is known about the nature of association between these polysaccharides and components of natural cell membranes. Therefore, a study of GAGs influence on membrane lipids is of particular importance. The aim of the present work is to get insight into the effect of hydrophilic dextran sulfate (DS)—that can be considered as GAG analogue—on membrane lipids organization. This study was based on examining interactions between DS sodium salt of molecular weight equal to about 40 kDa (DS40), dissolved in water subphase, and a model membrane, mimicked as Langmuir monolayer, formed by representative natural membrane lipids: cholesterol and 1,2-dipalmitoyl-sn-glycero-3-phosphocholine (DPPC) as well as their mixtures. Due to the fact that calcium ions in excess may accumulate in the lipid membrane, attracting high molecular weight molecules to their surface, the influence of calcium ions present in the subphase on the DS40 activity has also been examined. It has been found that negatively charged DS, forming a sublayer underneath the monolayer, barely interacts with membrane lipids; however, in the presence of calcium ions the electrostatic interactions between DS40 and lipid membrane are significantly enhanced, leading to the formation of network-like crystalline structures at the surface of model membrane, which can prevent incorporation and interaction with other extracellular molecules, e.g., proteins.

## Introduction

Glycosaminoglycans (GAGs) are essential components of the extracellular matrices (ECMs) that contribute to the stability, development, and communication of natural cells within all kinds of tissues (Bosman and Stamenkovic 2003; Saridaki et al. 2012). GAGs belong to the group of polysaccharides composed of long unbranched carbohydrate chains with repeating disaccharide units of an amino sugar (either *N*-acetylglucosamine or *N*-acetylgalactosamine) along with an uronic acid sugar (either glucuronic acid or iduronic acid) or galactose (Wight et al. 1991; Ruponen et al. 1999; Papy-Garcia et al. 2011). Although molecular structures of GAGs that naturally occur in living cells are rather uncomplicated, these polysaccharides can be subjected to further transformations, including enzymatic sulfonation of hydroxyl groups in carbohydrate moieties. As a result, a number of GAGs derivatives can be formed; both sulfated (including heparin and heparan sulfate, keratan sulfate, chondroitin sulfate, and dermatan sulfate) and non-sulfated (e.g., hyaluronic acid) (Lindahl and Li 2009; Papy-Garcia et al. 2011).

GAGs are primarily located on the outer surface of the cell membrane, being covalently attached to core proteins, forming proteoglycans (Sahoo and Schwille 2013). In smaller amounts, GAGs can also be found in the extracellular matrixes (ECMs) as free macromolecules. Hyaluronic acid—being an exception—does not form covalent linkages with membrane proteins, but interacts non-covalently with other ECMs molecules. The binding properties of the GAG chains are mainly determined by the substitution with the sulfo groups (Laabs et al. 2005), which results in highly negative charged structures. Therefore, GAGs are considered to be the strongest natural polyanions. Highly negative charge of the GAG chains induces binding of cations present within the ECM, what additionally ensures sufficient hydration and proper hydrostatic pressure inside tissues (Abaterusso and Gabaro 2006; Fukamil et al. 2007).

GAGs are involved in diverse biological processes acting on the surface and across natural cell membranes (Ruponen et al. 1999; Uygun et al. 2009; Papy-Garcia et al. 2011). Various scientific reports indicate a wide range of GAG application in the field of gene transfer. Recently, a lot of attention has been devoted to develop new methods in order to introduce genetic material into natural cells. On one hand, polyanionic GAGs interfere with gene transfer by binding to the positively charged complexes (e.g., DNA complexes). The resulting linkages can lead to changes in molecular charge and size of such complexes, affecting the cellular uptake. It was reported that GAGs, due to their ability to interact with cationic lipids, can bind to liposome/DNA complexes and thus initiate the release of DNA from the liposomes. On the other hand, GAGs mediate the binding of the cationic complexes to the surface of biological cells and in this way may act as important receptors for the cellular entry of the gene transfer complexes (Mislick and Baldeschwieler 1996; Ruponen et al. 1999).

GAGs also appear to play a significant role in the amyloid deposits in human pathologies (Ancsin 2003; Alexandrescu 2005; Smits et al. 2010; Saridaki et al. 2012). The amyloid formation is related to a protein folding disorder, which results in extracellular deposition of normally soluble peptides as insoluble fibrils, leading to the impaired tissue structure and its function.

Although GAGs seem to play an active role due to their high content in the extracellular matrix as well as their ability to interact with different types of proteins, their precise mode of action and induced effects still remain under debate. One of the hypotheses assumes that GAGs may act as pathological chaperones, which induce formation and stabilization of amyloid fibril. GAGs binding to amyloid fibrils, mainly through electrostatic interactions involving the negative polysaccharide charges and positively charged protein residues, may lead to abnormal accumulation of amyloids. This may result in the development of amyloidosis, being related—in brain—to the pathology of various neurodegenerative diseases, such as Alzheimer’s disease, Parkinson’s disease, and other prion-related diseases (Ruponen et al. 1999; Capila and Linhardt 2002; Bulow and Hobert 2004; Laabs et al. 2005). However, contradictory hypothesis implies that GAGs can inhibit amyloid formation by blocking the interactions of protein aggregates with cell membranes. Recent studies have shown that heparin does not directly bind or alter the structural and morphological properties of protein fibrils, pointing to its interaction with the cell surface. It is suggested that GAGs interact with natural cell membrane, probably competing with protein aggregates for the same binding sites on the cell surface (Saridaki et al. 2012; Lannuzzi et al. 2015). Taking into consideration that the presence of GAGs may significantly affect the structure and dynamics of biological cell membranes, it seems crucial to examine the potential impact of GAGs on membrane lipids.

The chemical complexity of the GAGs not only makes them less sensitive to conventional methods of analysis, but also leads to limitations in their isolation as well as fractionation. Thus, even fractionated and purified samples may contain a heterogeneous mixture of sequences of different lengths and compositions (Hallak et al. 2007). Considering these limitations, the analogues of GAGs, that can be obtained commercially in various average molecular sizes are often used instead of GAGs. Due to certain structural similarities, dextran as well as its derivatives can successfully replace cellular GAGs in experiments on model lipid membranes (Santos et al. 2005a, b, 2007a, b; Peetla et al. 2009). Most commonly applied dextran sulfate (DS) is an artificially sulfated polysaccharide produced by growing of *Leuconostoc mesenteroides* bacterial cultures. DS contains a high percentage of consecutive α(1,6)-glucosidic linkages and a relatively low percentage of α(1,3)-linked residues that makes the molecule stable and highly resistant to digestive enzymes, especially α-amylases, present in human body.

Dextran and its derivatives, considered as GAG analogues, were successfully applied to investigate various aspects of cellular interactions in lipid membranes. It has been shown that physicochemical properties of model GAGs can significantly influence the interactions with natural lipids as well as they can either increase or decrease the efficiency of drug delivery through the cellular membrane (Costin and Barnes 1975; Ross et al. 2001). On one hand, these rigid polysaccharides have the ability to inhibit undulation of lipid membranes. Experiments carried out on model black lipid membranes (BLMs) (Diederich et al. 1999) demonstrated that dextran derivative attached to the BLM surface significantly changes its mechanical properties. As a result, the increase of membrane viscosity and reduction of its stability was observed. On the other hand, model GAGs may decrease the energy barrier necessary to form a pore within lipid membrane, either by increasing the surface tension of the membrane or by reducing the edge energy of the pore (Diederich et al. 1999).

Intensive research on dextran and its derivatives—as model GAGs—has been found effective in the treatment of viral infections, in particular Sendai or HIV viruses, by inhibiting the entry of viruses into cells (Ramalho-Santos and Pedroso de Lima 2001; Wiethoff et al. 2001; Ruponen et al. 2004; Naessens et al. 2005). Moreover, these polysaccharides also seem to be promising in preventing the development of atherosclerosis in arteries and heart diseases (Nauck and Rifai 2000; Simons et al. 2002).

**Fig. 1:**
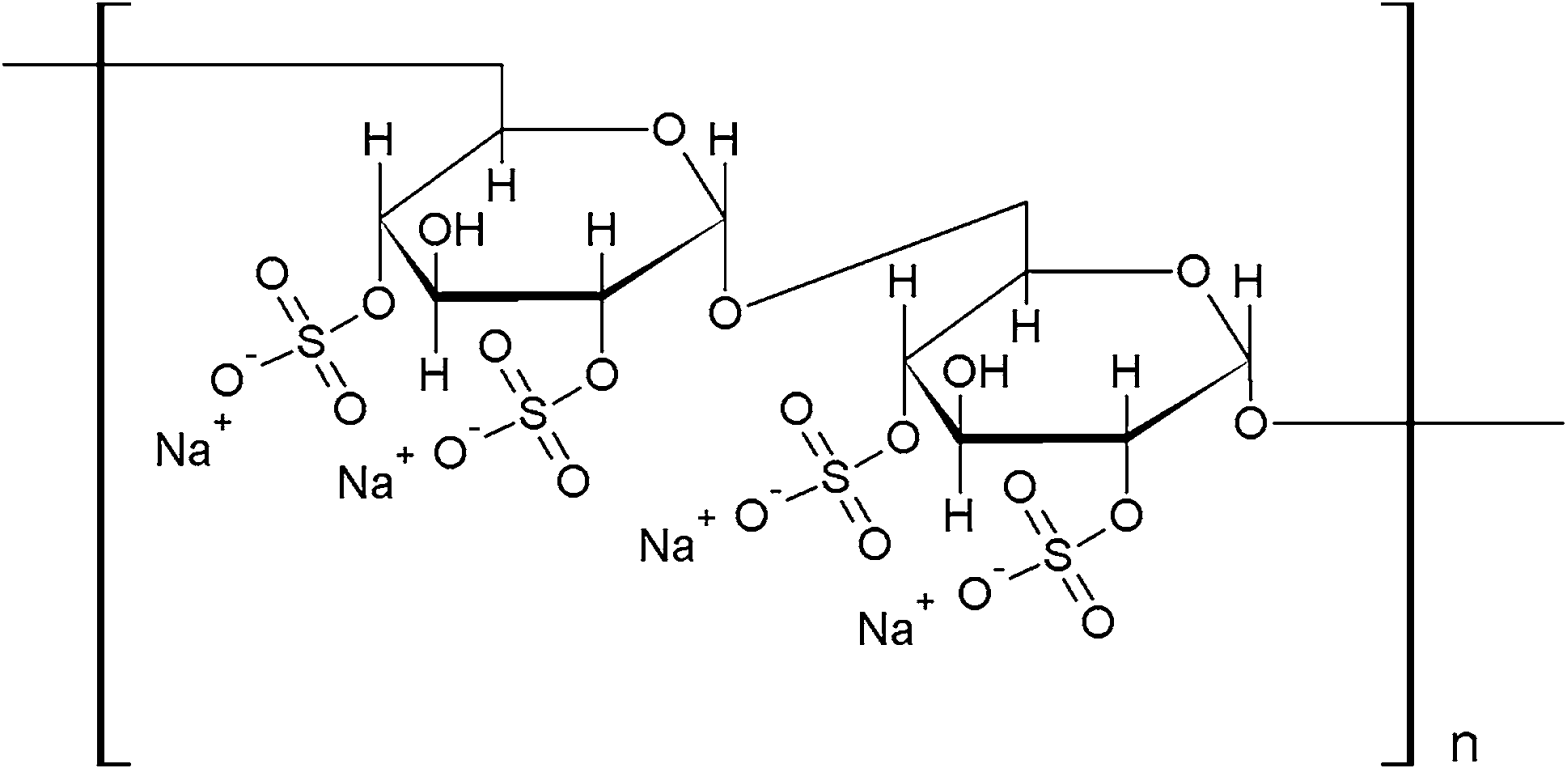
Chemical structure of DS sodium salt

Although the biophysical properties of DS have been studied rather well, the nature of its affinity to natural membranes has not been fully elucidated. Therefore, the aim of the present work is to examine the behavior of water-soluble DS of molecular weight equal to about 40 kDa (DS40) (Fig. 1) on model lipid environment. Since natural membranes are complex entities, in order to get insight into their interaction with biomolecules, model systems are usually applied, which are well-defined and simplified structures of natural membranes, and enable to study a particular aspect of biomolecule-membrane component interactions. In this respect, Langmuir monolayers are one of the most popular and successful membrane models (Maget-Dana 1999; Stefaniu et al. 2014; Nobre et al. 2015). With Langmuir monolayers, it is possible to easily construct a membrane of interest. The simplest model is a one-component monolayer, usually formed by one of the major membrane lipids, such as cholesterol, 1,2-dipalmitoyl-sn-3-phosphatidylcholine (DPPC) or other membrane component of interest. Such a model is certainly an oversimplification; however, it is useful in some cases, for example, it enables an easy verification of which of the membrane components are important in a particular aspect of its biological activity. More realistic are multicomponent Langmuir monolayers as membrane-mimicking systems, prepared by mixing of the representative components in an appropriate proportion found in natural systems. As the simplest model, mixed cholesterol/DPPC monolayer, mimicking an eukaryotic plasma membrane, is usually applied.

Taking the above into consideration, in this paper, we have studied the interactions between DS, penetrating into artificial membrane, mimicked as Langmuir monolayer, composed of representative lipids of mammalian cell membrane, such as DPPC, cholesterol, and their mixture. Since the increase of ionic strength or introduction of divalent cations are known to reduce the electrostatic repulsive forces between cell surfaces and thus may affect the interaction of DS40 with membrane lipids [which has been observed in vivo, showing that calcium ions in excess can accumulate at the lipid membrane, attracting high molecular weight molecules to its surface (Anderson et al. 1977)], the influence of Ca^2+^ on the DS40 activity has also been examined. The concentration of calcium ions used in our experiments (3 mmol dm^−3^) corresponds to its physiological content in the ECM.

## Experimental

### Materials

Cholesterol (> 99%) and DPPC (1,2-dipalmitoyl-sn-glycero-3-phosphocholine, synthetic, > 99%) were used as typical biomembrane lipids. Initially, each lipid was dissolved in a spectroscopic grade chloroform/ethanol (4:1 v/v) mixture made from chloroform (dedicated for HPLC, ≥ 99%) and anhydrous ethanol (> 99%). Then, the prepared solutions were spread onto the aqueous subphase in order to form insoluble films at the air/water interface. Stock solutions of cholesterol and DPPC were also applied to form mixed monolayers with different molar fractions of both constituents. Ultrapure water (from a Millipore system) and 4 × 10^−3^ mmol dm^−3^ solution of DS sodium salt with molecular weight of about 40 kDa (DS40) as well as DS40 solution enriched with calcium chloride (of the concentration 3 mmol dm^−3^) were used as subphases.

Cholesterol, DPPC, and DS40 were purchased from Sigma–Aldrich and used without further purification, while 3 mmol dm^−3^ calcium chloride aqueous solution was prepared from 0.05 mol dm^−3^ stock solution, manufactured by *POCh* (Poland).

### Methods

#### Langmuir Monolayer Technique

The surface pressure–area (π/*A*) isotherms were recorded by using a one-barrier Langmuir trough (NIMA, UK) of 300 cm^2^ total area. Surface pressure was measured with accuracy of ± 0.1 mN m^−1^ using a Wilhelmy plate made of ashless chromatography paper (Whatman Chr1). Before each measurement, water subphase was cleaned by closing the barrier and aspirating water until surface pressure readings were not exceeding ± 0.1 mN m^−1^, in comparison with values of surface pressure detected with the opened barrier. The subphase temperature was controlled thermostatically, by a circulating water system (Julabo), and kept at 20 °C ± 0.1 °C. Spreading solutions were deposited drop by drop onto the water subphase with a 250 µl Hamilton microsyringe, precise to 5.0 µl. After spreading, monolayers were left to equilibrate for 10 min and then compressed with barrier speed of 20 cm^2^ min^−1^. Each recorded π/*A* isotherm was repeated at least twice to ensure high reproducibility of the results.

#### Brewster Angle Microscopy (BAM)

Textures of the selected Langmuir monolayers were visualized with Brewster angle microscope, BAM (Accurion GmbH, Germany), equipped with a 50 mW laser emitting *p*-polarized light at a wavelength of 658 nm and an objective with tenfold magnification. The microscope was installed over a KSV NIMA Langmuir trough (Finland) with two barriers and 841 cm^2^ of its total area. BAM images show selected fragments of Langmuir monolayers with dimensions of 360 µm times 200 µm.

#### Langmuir–Blodgett Deposition Technique (LB)

Langmuir monolayers were transferred onto a solid surface (ruby muscovite mica of V-1 quality, purchased from *Continental Trade*), using LB technique. Before each experiment, mica substrate was placed, respectively, in the following subphases: water, aqueous solution of DS40 and DS40-containing Ca^2+^. After spreading solution of pure DPPC onto subphase, the monolayer was left to equilibrate for 10 min and then compressed to constant values of surface pressure (20 mN m^−1^). LB deposition was carried out by lifting-off the mica substrate from the relevant subphase through the monolayer with a dipper speed of 1 mm min^−1^ .

#### Atomic Force Microscopy (AFM)

Topographic images of the transferred films were recorded in air under ambient conditions using AFM (Agilent 5500) working in non-contact mode. The Al-coated force modulation silicon probes (Nanosensors) with spring constant about 2 N m^−1^, resonant frequencies about 80 kHz, and the tip radius equal to 7 nm were used. The set point and all gains were adjust to obtain minimal noise and high-quality images of examined surfaces. The topography images of each sample were recorded in several arbitrarily chosen locations. AFM data analysis was carried out using WSxM free software (Horcas et al. 2007).

## Results and Discussion

In order to determine the effect of DS40 on model cellular membrane, in the first step of our investigations, we have examined its influence separately on two major membrane lipids: cholesterol and DPPC, and subsequently on cholesterol/DPPC mixed monolayers as a simplified biomembrane model. Although the behavior of the above membrane lipids and their mixtures have already been studied in detail (Cadena-Nava et al. 2006; Sabatini et al. 2008; Miñones et al. 2009), we present the π/*A* isotherms and BAM images recorded on water subphase for the purpose of comparison with the results obtained for DS40 and/or Ca^2+^-containing subphases.

### π/*A* Isotherms and BAM Images for Pure Lipids Monolayers on Different Subphases

**Fig. 2:**
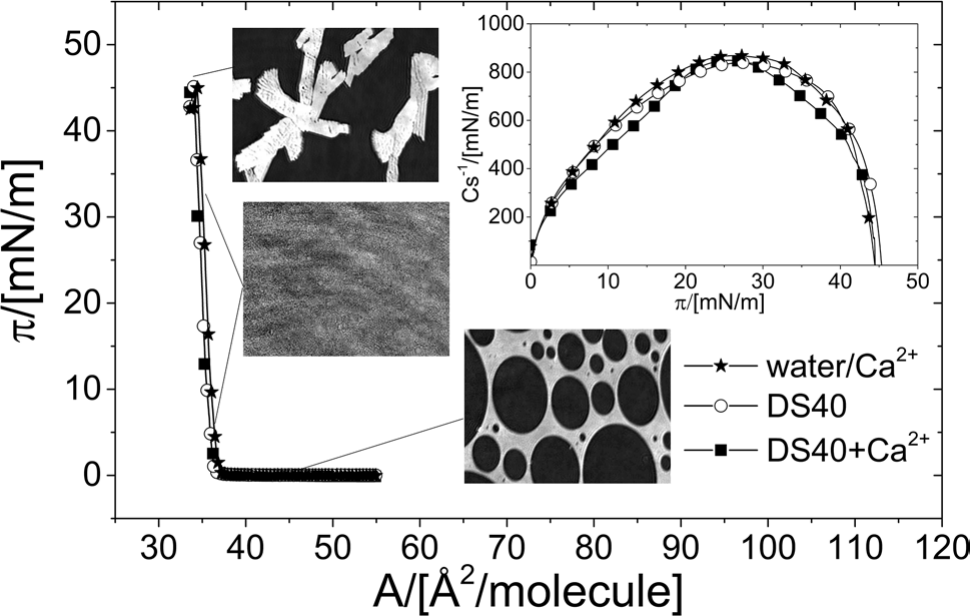
π/*A* isotherms for cholesterol monolayers, formed on the following subphases: water, water-containing Ca^2+^, aqueous solution of DS40, and DS40-containing Ca^2+^ at 20 °C. Inset: compression moduli (*C*_s_^−1^) values as a function of surface pressure

Surface pressure–area (π/*A*) isotherms registered for pure cholesterol monolayers formed on different subphases at 20 °C, complemented with the values of compression moduli (*C*_s_^−1^), illustrated as a function of surface pressure (π), complemented with selected BAM images are presented in Fig. 2.

**Fig. 3:**
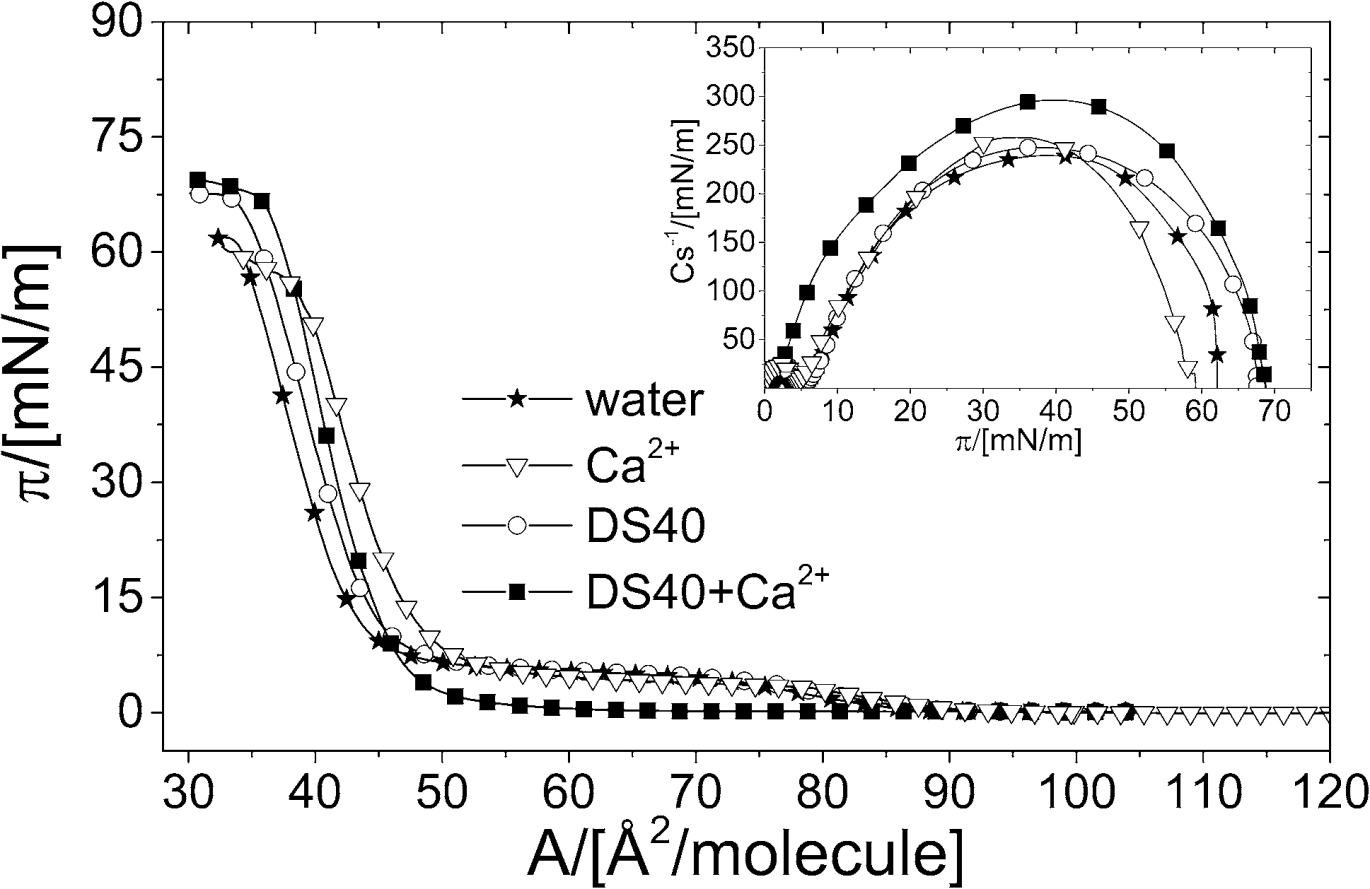
π/*A* isotherms for DPPC monolayers, formed on the following subphases: water, water-containing Ca^2+^, aqueous solution of DS40, and DS40-containing Ca^2+^ at 20 °C. Inset: compression moduli (*C*_s_^−1^) values as a function of surface pressure

The results recorded for water subphase are in good agreement with those presented elsewhere (Cadena-Nava et al. 2006). The course of the obtained π/*A* isotherms indicates that cholesterol forms a highly condensed monolayer in the form of a typical solid film (S) on all the applied subphases, which can be also confirmed by high compression moduli values (called also “surface elasticity”, defined as ${C_s}^{{ - 1}}=A{\left( {\partial \uppi /\partial A} \right)_{\text{T}}}$ ; *A* denotes average area per lipid molecule in a monolayer) (Davies and Rideal 1963) (Fig. 2). The introduction of DS40 and/or Ca^2+^ to the subphase practically does not affect both the shape and the position of the isotherm from cholesterol. The observed difference in the collapse pressure (π_c_) value for cholesterol monolayer spread on DS40+Ca^2+^ (47 mN m^−1^) as compared to other investigated here subphases (π_c_ ~ 45 mN m^−1^) is negligible. The visualization of cholesterol monolayer with BAM (Fig. 2) does not reveal any significant differences in the films texture on different subphases investigated. Therefore, it can be concluded that the introduction of DS40 and/or Ca^2+^ into water subphase has no impact on the organization as well as packing arrangement of sterol molecules. For comparison, similar experiments were performed with another important membrane lipid: DPPC (Fig. 3).

The shape of π/*A* isotherm registered for pure DPPC monolayer on the water subphase is very similar to that recorded on aqueous DS40 or Ca^2+^ solutions, with a characteristic plateau at 5 mN m^−1^, which have been ascribed to a phase transition between liquid-expanded and liquid-condensed (LE–LC) states (Miñones et al. 2009). This plateau is visible as a minimum on *C*_s_^−1^ versus π plots for pure DPPC monolayer formed both on water, water-containing Ca^2+^, and aqueous DS40 solution (Fig. 3, inset). A striking difference is the disappearance of plateau region for DPPC on DS40-containing Ca^2+^ solution. This is associated with monolayer condensation as evidenced by higher *C*_s_^−1^ values as compared to DPPC films spread on other investigated solutions. Moreover, it is worth noticing that stability of films formed on DS40-containing subphases increases as shown by higher collapse pressure values.

**Fig. 4:**
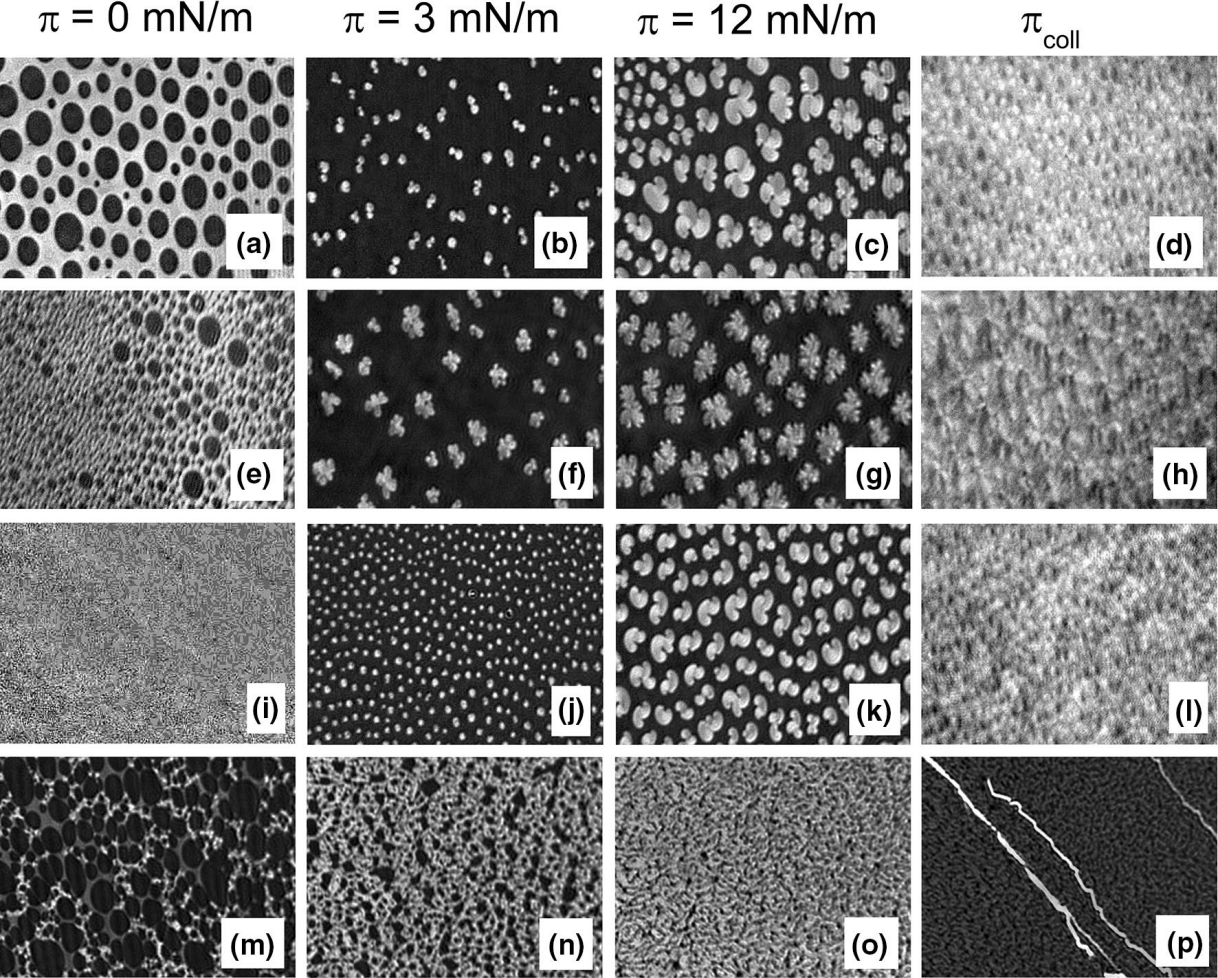
BAM images registered at selected values of surface pressure for DPPC monolayers, formed on the following subphases: water (**a**–**d**), water-containing Ca^2+^ (**e**–**h**), aqueous solution of DS40 (**i**–**l**) and DS40-containing Ca^2+^ (**m**–**p**) at 20 °C

Despite close similarities between π/*A* isotherm registered for DPPC monolayer on water and on aqueous Ca^2+^ or DS40 solution, there are visible differences in the morphology of the studied systems (Fig. 4). Accurate analysis of films textures with BAM shows changes in the shape of domains formed at the surface, particularly well distinguishable just above the plateau region (~ 12 mN m^−1^). Namely, for DPPC monolayers spread on water, the domains show characteristic ‘clover’ shape, while upon addition of Ca^2+^ ions their shape is changed to the ‘flower’-like, which is in agreement with literature (Camara and Wilke 2017). On aqueous DS40 solution domains morphology is changed to the ‘S’-shaped structures. It is well known that DPPC molecules in a broad pH range (2.1–13.9) occur in the electroneutral (zwitterionic) form (Fisar 2005). This means that at physiological pH of the extracellular space (7.35–7.45) phospholipid molecules comprise an equal number of ionized groups with opposite charge. The negative charge is localized on the oxygen atom of the phosphate group, while a positive one—on the nitrogen atom of the choline group. Stability of DPPC monolayer is maintained by lateral repulsions between film molecules. Upon introduction of calcium ions, the negatively charged phosphate groups of DPPC molecules interact electrostatically with Ca^2+^, turning the zwitterionic monolayer to cationic, without any pronounced changes in the isotherm characteristics. For DPPC spread on DS40 solution, the polyelectrolyte brings in an additional negative charge due to the formation of a sublayer of DS40 strains underneath the phospholipid film as proved elsewhere (Santos et al. 2005b; Camara and Wilke 2017). This negative charge is reduced by the introduction of calcium ions into aqueous dextran sulfate solution as bivalent ions contribute to electrostatic interactions between the anionic phosphate groups of DPPC and the anionic polysulfate groups of DS40 by the formation of calcium bridges. In consequence, DPPC/Ca^2+^/DS40 complex is formed, resulting in strong adsorption of DS40 to the surface, already confirmed in literature (Santos et al. 2005b), and condensation of DPPC monolayer, which is reflected both in higher *C*_s_^−1^ values and disappearance of liquid-expanded to liquid-condensed transition. In consequence, in BAM images characteristic for this region domains are replaced with network-like crystalline structures.

**Fig. 5:**
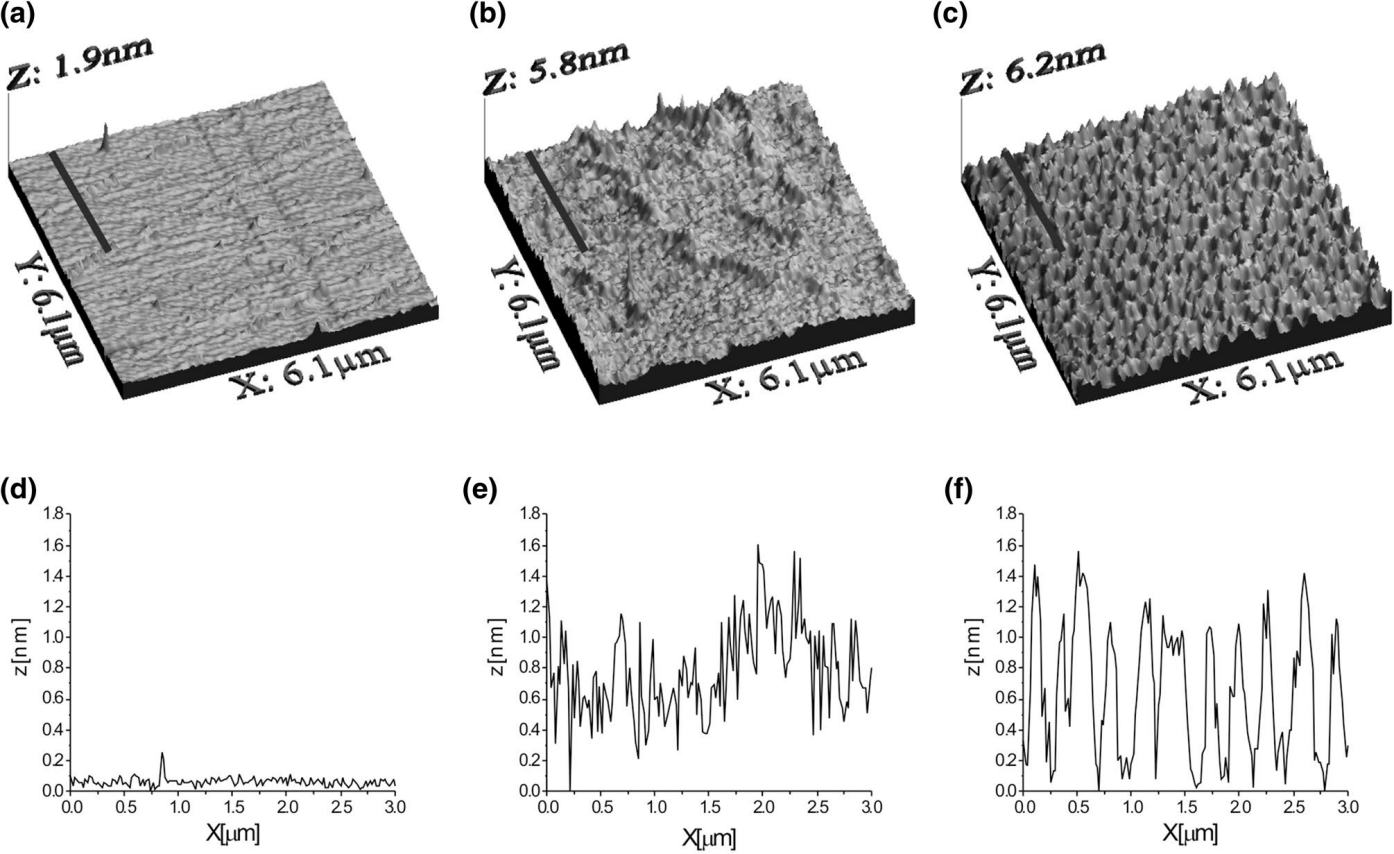
AFM images of **a** DPPC monolayer transferred from a water subphase, **b** DPPC film transferred from an aqueous solution of DS40, and **c** coupled DPPC/Ca^2+^/DS40 complex, deposited on mica substrates at the surface pressure of 20 mN m^−1^ using LB technique, complemented with height profiles from highlighted lines (**d, e**, and **f**, respectively)

In order to characterize the observed structures in detail, we have transferred DPPC/Ca^2+^/DS40 complex to solid support, using the LB technique. For comparison, DPPC monolayers spread on water and aqueous DS40 solution have also been transferred. The obtained AFM images deposited on mica substrates are presented in Fig. 5a–c.

The AFM image of DPPC monolayer transferred from water subphase (Fig. 5a) shows uniform and homogenous structure with minimal height or compliance variations as reflected by the root mean square (RMS) roughness parameter equal to 0.14 nm. Similar structure for DPPC monolayer deposited on mica substrate has already been presented in literature (Kim et al. 2013). The surface of DPPC film transferred from an aqueous DS40 solution is characterized by a slightly higher RMS roughness parameter reaching up to 0.3 nm; however, the homogeneity of the observed surface is preserved (Fig. 5b). The AFM image of DPPC/Ca^2+^/DS40 complex (Fig. 5c) shows continuous layer with clearly visible gaps (RMS parameter equal to 0.37 nm), confirming the formation of complex, which structure was found to depend on the amount of adsorbed DS40 (Huster 1998).

### π/*A* Isotherms for Cholesterol/DPPC Mixed Monolayers on DS-Containing Subphases

**Fig. 6:**
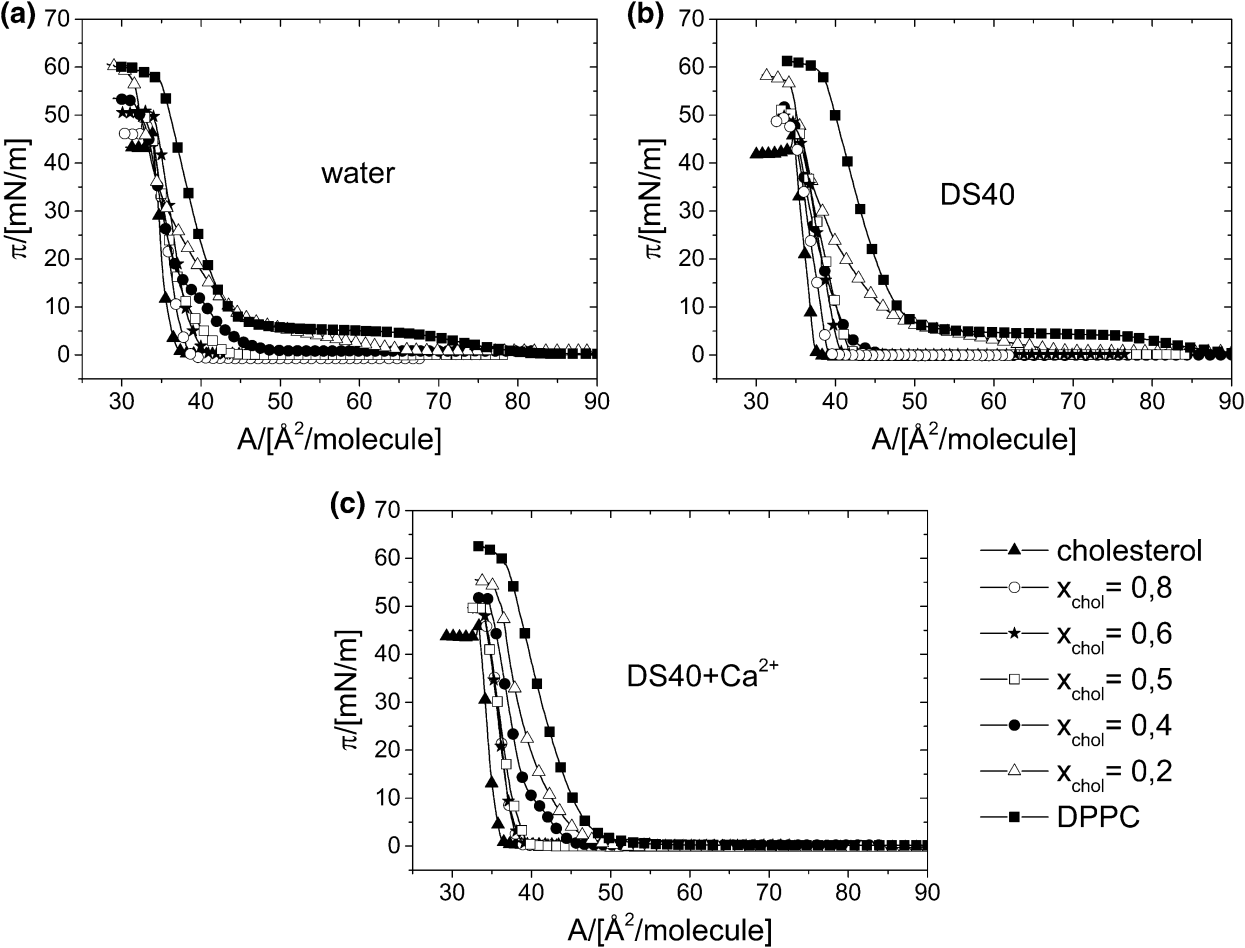
π/*A* isotherms for cholesterol/DPPC mixed monolayers, formed on the following subphases: **a** water, **b** aqueous solution of DS40, and **c** DS40-containing Ca^2+^ at 20 °C

π/*A* isotherms for cholesterol/DPPC mixed monolayers, formed on different subphases at 20 °C, are presented in Fig. 6a–c. Although this system, considered as the simplest model of cell membrane, has been widely investigated (see for example: Wydro and Hąc-Wydro 2007), the monolayer results obtained on water are included in this paper for the purpose of comparison with those obtained on DS-containing subphases.

It is seen that upon increasing of cholesterol content in DPPC monolayers for all the studied subphases, the isotherms are shifted towards lower values of the average area per lipid molecule (proving the so-called “condensing effect” of cholesterol). Such a behavior of area condensation is typical for phospholipid–cholesterol mixtures and has been widely described in literature (Sabatini et al. 2008).

**Fig. 7:**
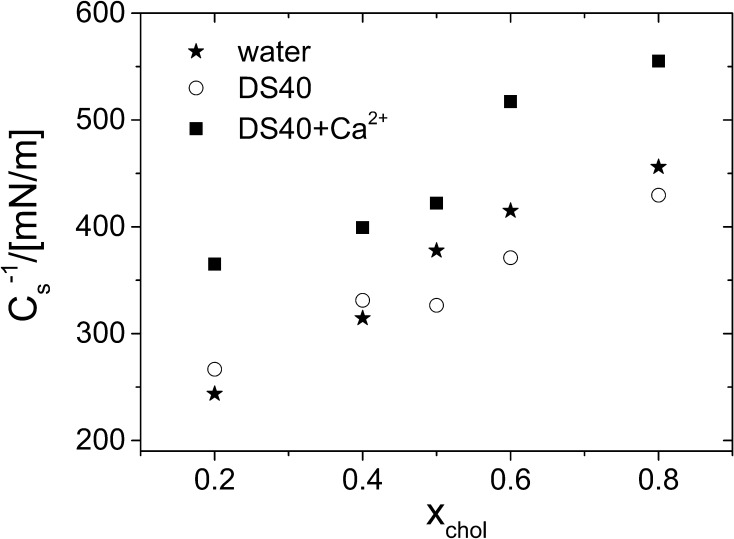
The maximum values of compression moduli (*C*_s_^−1^) due to incorporation of cholesterol into mixed monolayers, at 30 mN m^−1^

The comparison of maximum values of the compressibility moduli (*C*_s_^−1^), presented in Fig. 7, clearly demonstrates that the change of water subphase to DS40 solution practically does not influence their elasticity, while upon the addition of calcium ions the monolayers become more rigid (higher *C*_s_^−1^ values).

**Fig. 8:**
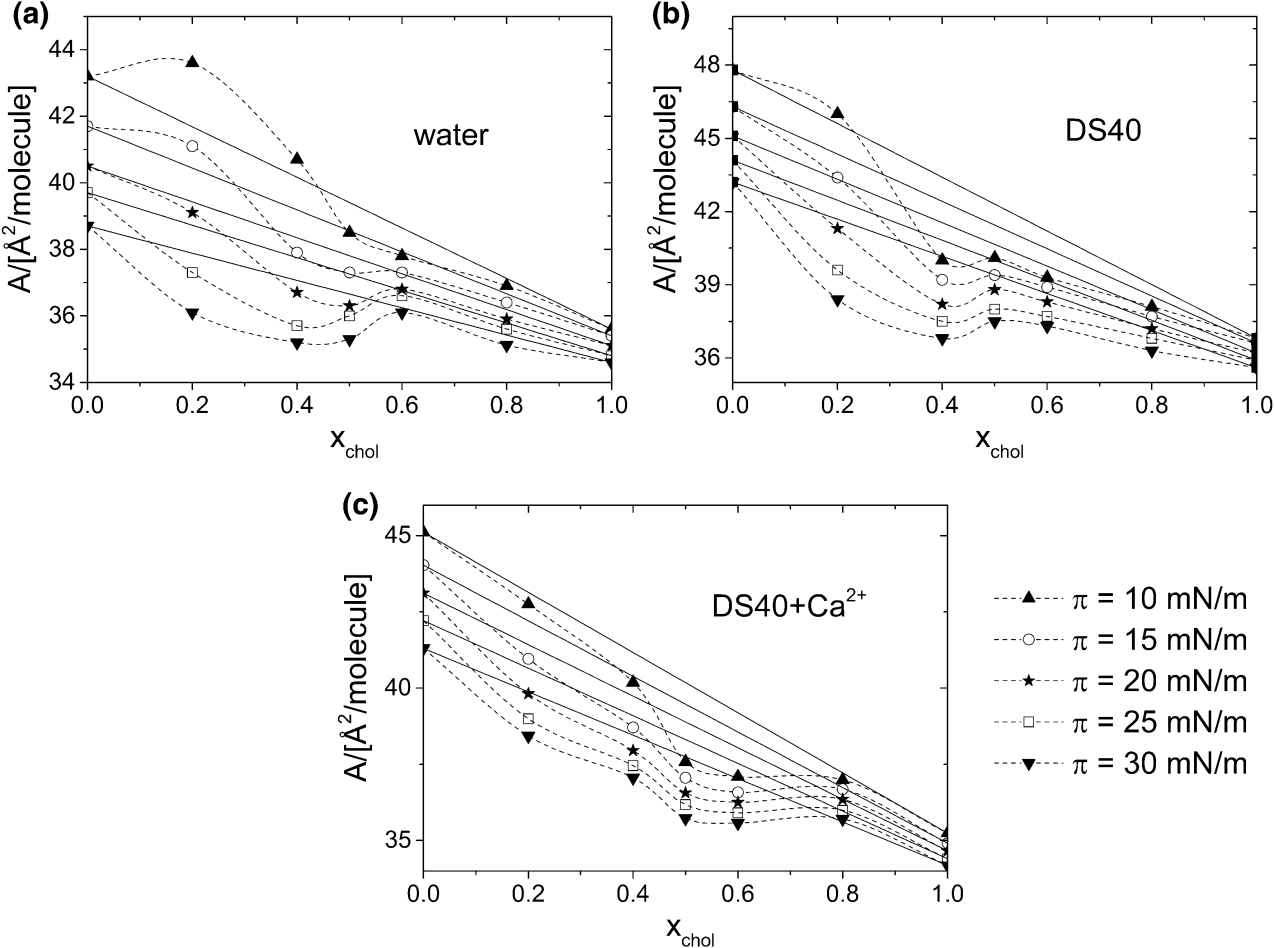
Values of mean molecular area (*A*_12_) as a function of cholesterol molar fraction for cholesterol/DPPC mixed monolayers, formed on the following subphases: **a** water, **b** aqueous solution of DS40, and **c** DS40-containing Ca^2+^ at 20 °C

To get insight into the interactions on different subphases, the plots of the average area per molecule (*A*_12_*)* in mixed monolayers as a function of cholesterol molar fraction (at different constant values of surface pressures: 10, 15, 20, 25, 30 mN m^−1^) were drawn from experimental data points of isotherms (Fig. 8). The obtained curves, presented in Fig. 8a–c, allow to conclude about interactions and miscibility of the film components. If either an ideal mixed monolayer is formed or two components are completely immiscible, the additivity rule is obeyed (Dynarowicz-Latka and Kita 1999):

1 $${{\text{A}}_{{\text{12}}}}={{\text{X}}_{\text{1}}}{{\text{A}}_{\text{1}}}+{{\text{X}}_{\text{2}}}{{\text{A}}_{\text{2}}},$$

where *A*_1_, *A*_2_ are defined as the average area per lipid molecule in pure monolayers and *X*_1_, *X*_2_ correspond to their molar fractions in mixed film, and the plots presenting the average area per lipid molecule as a function of molar fraction form a straight line (dotted lines in Fig. 8a–c). Any deviations from the straight indicate miscibility and non-ideality. Considering the results for cholesterol/DPPC system, apparent deviations from ideality are observed. This clearly proves the miscibility of cholesterol and DPPC in monolayers spread on all the studied subphases.

For miscible system, it is possible to perform quantitative analysis of interactions, which can be done with the values of the excess free enthalpy of mixing (Δ*G*^ex^) plotted as a function of cholesterol molar fraction (at different constant values of surface pressure: 10, 15, 20, 25, 30 mN m^−1^), calculated according to the following Eq. (2) (Gaines 1966; Costin and Barnes 1975):

2 $$\Delta {{\text{G}}^{{\text{exc}}}}={\text{N}}\int\limits_{{\text{0}}}^{{{{\uppi}}}} {{\text{(}}{{\text{A}}_{{\text{12}}}} - {{\text{X}}_{\text{1}}}{{\text{A}}_{\text{1}}} - {{\text{X}}_{\text{2}}}{{\text{A}}_{\text{2}}}{\text{)d}}\uppi },$$

wherein *N* is the Avogadro number.

**Fig. 9:**
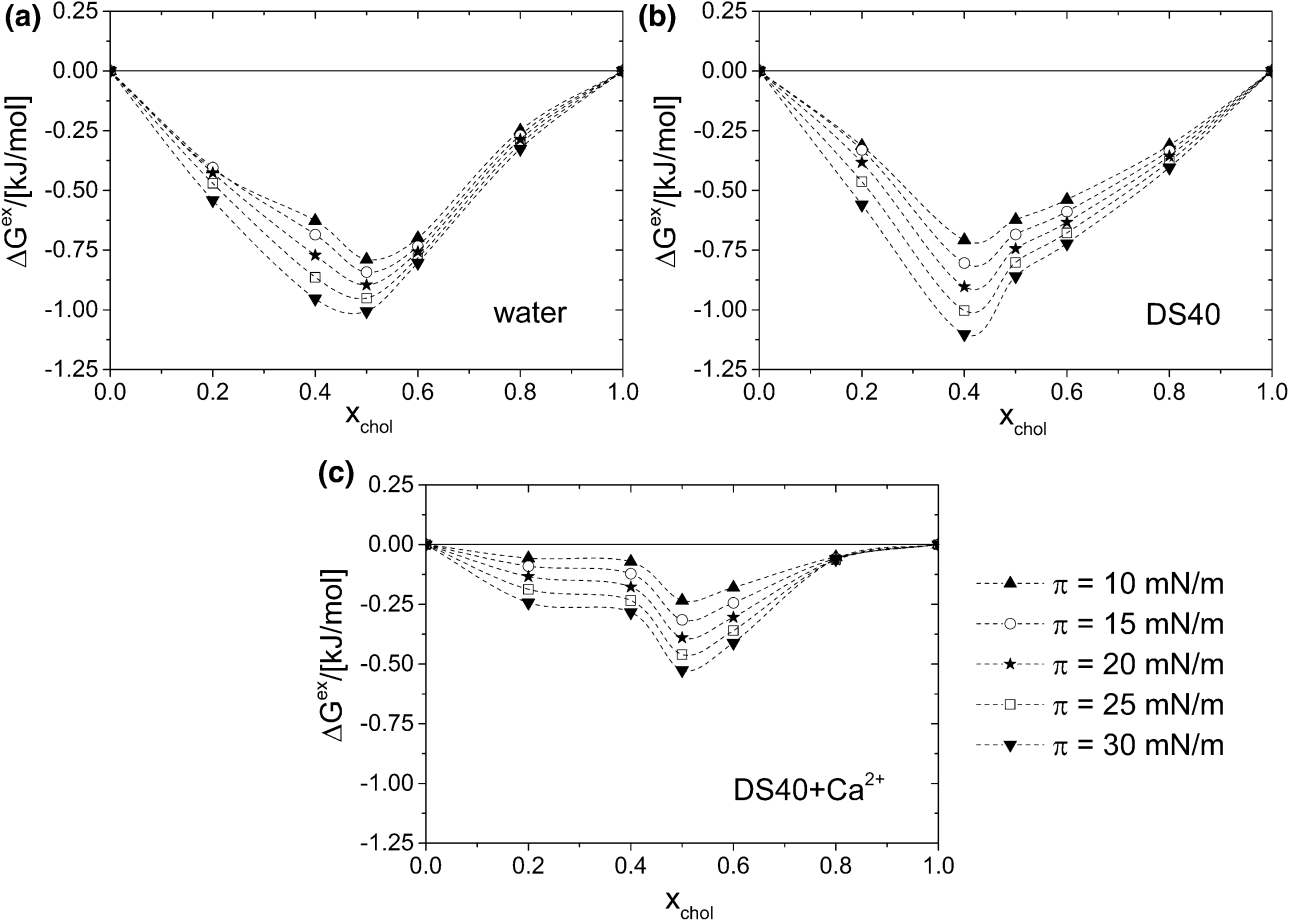
Values of the excess free enthalpy of mixing (Δ*G*^exc^) as a function of cholesterol molar fraction for cholesterol/DPPC mixed monolayers, formed on the following subphases: **a** water, **b** aqueous solution of DS40, and **c** DS40-containing Ca^2+^ at 20 °C

The obtained dependencies, presented in Fig. 9a–c, show that the strength of cholesterol–DPPC interactions depends on the kind of the applied subphase.

Considering films formed on water, negative values of Δ*G*^ex^ are observed, indicating the existence of strong interactions between film-forming molecules. The minimum value of Δ*G*^ex^ corresponds to the mixture of the strongest interactions and maximum thermodynamic stability, which occurs at X_chol_ ~ 0.5 (Wydro and Hąc-Wydro 2007). In literature, this phenomenon has been interpreted as being due to stable cholesterol–DPPC complex formation of 1:1 stoichiometry (Brzozowska and Figaszewski 2002; Dynarowicz-Latka et al. 2002; Sabatini et al. 2008) due to the hydrogen bond formation between molecules. Upon introducing of DS40 into the subphase, the strength of interactions remains similar as evidenced by comparable maximum Δ*G*^ex^ values on both subphases. However, the additionally introduced calcium ions have a considerable impact on DS40 interaction with model lipid membrane, which is seen in less negative Δ*G*^ex^ values (that change from −1 kJ mol^−1^ at the minimum for water/DS40 solution to – 0.5 kJ mol^−1^ for DS40 + Ca^2+^). From the thermodynamic point of view this means that bivalent ions exert unfavorable effect on DPPC/cholesterol monolayers on DS40 solution. Such thermodynamic destabilization of the monolayers results from weakening of the DPPC–sterol interaction by the formation of calcium bridges between DPPC and DS40. Moreover, additional effect of conformational changes in the presence of calcium ions of the phosphatidylcholine headgroup, which has been reported (Simon et al. 1975; Akutsu and Seelig 1981; Ross et al. 2001), can also account to the magnitude of interactions.

## Conclusions

The results of our experiments on the effect of DS or/and Ca^2+^ ions on major membrane lipid monolayers show no effect on cholesterol while pronounced changes have been observed for DPPC monolayer. Both plateau transition and elasticity of monolayer are altered in the presence of divalent ions in the DS subphase, accompanied by significant changes in the morphology of monolayer, showing network-like crystalline structures, associated with the formation of calcium bridges between phosphate groups of DPPC and polysulfate groups of DS. The results of our experiments also evidenced that DS in the presence of Ca^2+^ has a significant influence on model biological membrane. Hydrogen bonds between cholesterol and DPPC in mixed monolayers are weakened in the presence of Ca^2+^, due to electrostatic interactions with negatively charged groups of DPPC and DS. In this way, DPPC–cholesterol monolayers are destabilized as proved with less negative Δ*G*^ex^ values. Moreover, they lose their flexibility and become more rigid. Therefore, the formation of calcium bridges can be of biological importance as their presence prevents DS from interacting with other extracellular molecules, for example, proteins, and hinders the possibility of incorporation of other solutes. Strong interactions that occur between DS and membrane lipids *via* calcium ions may also be of great importance in discussing a possible role of ECMs for lipid dynamics in cell membranes, which are essential processes in cellular signaling.
